# Temperature and duration of exposure drive infection intensity with the amphibian pathogen *Batrachochytrium dendrobatidis*

**DOI:** 10.7717/peerj.12889

**Published:** 2022-02-07

**Authors:** Jon Bielby, Cristina Sausor, Camino Monsalve-Carcaño, Jaime Bosch

**Affiliations:** 1 School of Natural Sciences and Psychology, Liverpool John Moores University, Liverpool, United Kingdom; 2 Museo Nacional de Ciencias Naturales, CSIC, Madrid, Spain; 3 TRAGSATEC, Madrid, Spain; 4 Biodiversity Research Institute (University of Oviedo-CSIC-Principality of Asturias), Mieres, Spain

**Keywords:** Alyes obstetricans, Chytridiomycosis, Overwintering larvae, Gosner

## Abstract

The intensity of a pathogen infection plays a key role in determining how the host responds to infection. Hosts with high infections are more likely to transmit infection to others, and are may be more likely to experience progression from infection to disease symptoms, to being physiologically compromised by disease. Understanding how and why hosts exhibit variation in infection intensity therefore plays a major part in developing and implementing measures aimed at controlling infection spread, its effects, and its chance of persisting and circulating within a population of hosts. To track the relative importance of a number of variables in determining the level of infection intensity, we ran field-surveys at two breeding sites over a 12 month period using marked larvae of the common midwife toad (*Alyes obstetricans*) and their levels of infection with the amphibian pathogen *Batrachochytrium dendrobatidis* (Bd). At each sampling occasion we measured the density of larvae, the temperature of the water in the 48 h prior to sampling, the period of time the sampled individual had been in the water body, the developmental (Gosner) stage and the intensity of Bd infection of the individual. Overall our data suggest that the temperature and the duration of time spent in the water play a major role in determining the intensity of Bd infection within an individual host. However, although the duration of time spent in the water was clearly associated with infection intensity, the relationship was negative: larvae that had spent less than 3–6 months in the water had significantly higher infection intensities than those that had spent over 12 months, although this infection intensity peaked between 9 and 12 months. This could be due to animals with heavier infections developing more quickly, suffering increased mortality or, more likely, losing their mouthparts (the only part of anuran larvae that can be infected with Bd). Overall, our results identify drivers of infection intensity, and potentially transmissibility and spread, and we attribute these differences to both host and pathogen biology.

## Introduction

The effect of a pathogen on an individual host and that of its population varies depending on a number of biotic and abiotic factors (Lachish et al., 2011; Clark et al., 2016). One way the impact of a pathogen may be altered is *via* the number of infectious bodies or particles with which a host is infected (hereafter ‘infection intensity’). This parameter may vary greatly among individuals within a population of hosts, and can be important in a number of ways. For example, infection intensity can be a key determinant in which individuals are most likely to spread infection (Lloyd-Smith et al., 2005; Beldomenico, 2020), and the impact of the pathogen on the health of that individual host (Beldomenico & Begon, 2010).

In a notable amphibian pathogen, *Batrachochytrium dendrobatidis* (hereafter *Bd*), evidence suggests that infection intensity is one of a number of epidemiological parameters, including prevalence of infection, that is positively associated with pathogen transmission (Vredenburg et al., 2010; Tompros et al., 2021). This generalist pathogen is known to infect multiple hosts around the world, and its effects are known to vary both inter- and intra-specifically (Rosa et al., 2017), in part as a result of infection intensity (Whitfield et al., 2017). The role of infection intensity can also be complicated by non-linearities in how the variable changes over time and relates to individual and population-level effects. For example, there can be circularity between infection intensity and ill-health: that is, how and when does high infection intensity cause ill-health, and when does ill-health predispose an individual to higher levels of infection (Beldomenico & Begon, 2010)? Gaining better knowledge on the drivers of infection levels and their complex interactions may help us to better predict and mitigate both individual and population-level outcomes of pathogen exposure.

Important drivers of infection intensity include variation in immune response (Nava-González et al., 2021), individual health status and susceptibility (Beldomenico & Begon, 2010; Brunner et al., 2017), behavioural patterns (Burrowes et al., 2017) and the environment in which infection occurs (McMillan et al., 2020; Carter et al., 2021). Another reason for variation in infection intensity is heterogeneity in how pathogen exposure occurs and for how long it lasts (Kumar et al., 2020). Variation in risk of infection (or reinfection) can be broken down into a smaller set of constituent parameters, which form the basis of epidemiological theory and practice: the duration of infection (and infectiousness); the contact rate between individuals within the population; and the probability of transmission for a given contact between individuals.

The duration of infection and infectiousness determines the time period over which an individual may transmit a pathogen to other individuals and over which infection may proliferate within that host (Brunner et al., 2004). For amphibians and *Bd*, this duration may also vary according to the species in question, its life-history strategy within that population, and the life-stage of the species. For example, in pond breeding amphibians there can be great variation in how long different species and life-stages of those species spend in and around the water. The differences in life history strategy that host age categories exhibit can help to explain a great deal about the roles that different hosts play in the maintenance and spread of pathogens (Bielby et al., 2021). Some species have extended larval stages that may last for 4 or 5 years, including overwintering within the pond. The tendency to remain aquatic for an extended period has been linked to maintenance of pathogen infection, and to which species are likely to act as a reservoir of infection and potentially suffer long-term negative impacts of infection (Brunner et al., 2004; Medina et al., 2015; Fernández-Beaskoetxea, Bosch & Bielby, 2016; Bielby et al., 2021).

The number of contacts between individuals within a population is another key parameter in understanding infection intensities within a population. All else being equal, a higher rate of contact will lead to a great number of opportunities to become infected or re-infected (Rachowicz & Briggs, 2007). Contact rates may not simply increase as a linear function of density (Fenton et al., 2002; Rachowicz & Briggs, 2007; Malagon et al., 2020), and density and frequency-dependent transmission are common modes of transmission for *Bd* and other infectious pathogens. These modes suggest that the density of hosts may affect transmission rates in very different ways, highlighting the importance of measuring this parameter when trying to understand infection transmission. The abundance of amphibians over the course of a year is therefore a relatively simple metric to quantify in the absence of detailed data on actual contact rates.

Infection intensity within a host will also vary according to *Bd* life history at a given temperature, and the immune response of the host, both of which may interact to determine the probability of transmission for a given contact. Both are significantly affected by the seasonality of the particular community of amphibians they infect. While the thermal optimum of *Bd*, at least *in vitro*, has been measured to be 17–25 °C (Piotrowski, Annis & Longcore, 2004), the pathogen has been shown to alter its life history in response to changes in temperature (Woodhams et al., 2008) and may exhibit local adaptation as a result (Voyles et al., 2012; Stevenson et al., 2013). Further, amphibian immune response can alter considerably in response to temperature change (Raffel et al., 2006; Ribas et al., 2009). While ectotherms may be able to modify their immune response when temperature changes, the time-lag involved is often considerable, certainly taking longer than the infection dynamics of many pathogens, *Bd* included (Raffel et al., 2006). Further, the larval stages of many amphibians are known to play a key role in the maintenance and spread of infection (Brunner et al., 2004; Bielby et al., 2021), but they mount little immune response in those parts of the individual that harbour *Bd* infection: larval anuran amphibians are only infected in their keratinous mouthparts. The immune response of the larvae and their mouthparts are functionally less well developed than those of metamorphic individuals, and infection here is not usually fatal (Grogan et al., 2018). Interactions between temperature and infection intensity may therefore vary with species, life-stage and age-class, but there is a sound basis for predicting that water temperature will play a key role in driving infection levels and infection transmission.

In this study we use season-long data on two close populations of the common midwife toad, *Alytes obstetricans*, to try to understand drivers of infection intensity of the generalist pathogen *Bd*. Common midwife toads are a suitable study species as they are known to play an important role in the transmission and persistence of *Bd* (Bielby et al., 2021), and the species has shown significant variation in its response to *Bd* exposure and infection across its range (Tobler & Schmidt, 2010). In part, the importance of *A. obstericans* as a *Bd* host is due to the developmental phase of its larvae, the speed of development of which can vary greatly depending on their elevation. For example, populations at sea-level will typically develop in their hatching year, whereas those at higher elevations always overwinter, sometimes for multiple years (>~1,500 m; Bosch, 2002). However, at intermediate elevations both of these strategies may be exhibited. Typically the time spent in water is strongly associated with the developmental stage of amphibian larvae; that is, the longer the time spent in the water, the higher the Gosner stage. However, depending on the life-history strategy of the populations in question, Gosner stages may have spent very different amounts of time in the water body exposed to *Bd*. For example, some eggs may hatch in spring and complete metamorphose in a single season (4–6 months). In contrast, some clutches may be laid and hatch in late summer. These will overwinter as larvae, and will develop and metamorphose early the following season in early summer (around June). This variation in speed of development within a population provides a unique opportunity to investigate the relative importance upon infection intensity of the time an individual larva has spent in the water compared to its Gosner stage, as the two are independent of one another. This is important if we want to understand how physiology (*i.e*. Gosner stage and changes in keratin availability) and duration of pathogen exposure (*i.e*. time spent in the waterbody) vary and interact to explain the patterns of infection that we observe.

We use quantitative data on *Bd* infection burden taken at four time-points throughout the year to investigate our hypothesis that individuals will have variation in infection intensity, and that this variation is driven by a number of biotic and abiotic predictors. While infection intensity, as measured by *Bd* copies count obtained from qPCR, is not without its issues (Clare et al., 2016), we suggest that it is suitable for identifying how broad categories that may vary in their effect on infection level. Further, given the non-lethal, repeated measures taken in this study, and the higher sensitivity of qPCR over other methods (*e.g*. histology), qPCR are a powerful and suitable method for answering our questions. Specifically, here we suggest that infection intensity should vary in accordance with the following predictions: P1: Infection intensity will be higher when the density of hosts is highest, as contact rates between individuals and number of *Bd* zoospores in the water body will be elevated; P2: After a period of lower water temperature infection intensity will increase, as amphibian immune response is likely to be sub-optimal, while *Bd* may be more suited to changes as a result of local-adaptations; P3: Individuals that have been in the water body, and therefore have been exposed to *Bd* for the longest will have higher infection intensities; P4: Finally, we would predict that the Gosner stage of the larvae would be positively associated with infection intensity up until metamorphosis, because the more advanced the development of the larva, the longer it has been exposed to infectious zoospores in the water body and the more keratin it should have throughout its body. This final prediction is distinct from P3 in that although linked, Gosner stage and time in the water are not always perfectly correlated. Hence, it is important to include both in the analyses with separate predictions to address the question of whether it is developmental stage or time in the water body (or both) that dictate the infection level of an individual larva.

## Materials and Methods

The study area is located in the municipality of Toro (Zamora province, Castilla y León, Spain), where very old artificial troughs for cattle are abundant. Common midwife toads breed in these troughs, and may deposit egg clutches at two separate times of the year: some clutches are deposited in spring (March–April), while some clutches are released in autumn (J. Bosch, 2014–2021, personal observations). Larvae from autumn eggs will typically overwinter. This unique system means that within a single calendar year, larvae hatched at different times will be present at a single sampling visit. The existing classes of larval ages included in sampling were overwintered larvae, newly hatched larvae, young of year larvae (*i.e*. no longer newly hatched, but not having overwintered), and metamorphic individuals. At the first April sampling overwintering larvae (OW) remained from the previous year and new hatched larvae (NH) were also present. In the July sampling, any OW larvae that were present in April would have completed their metamorphosis (MM), and any NH that were present in April will have progressed and would now be classified as young of the year larvae (YOY) to distinguish them from July’s NH. In October, the YOY from July would have metamorphosed (MM), and NH from July would have become YOY larvae, with new October NH also being present. Finally, in January only OW larvae are present. No other species of amphibians have been found to use these troughs.

Two troughs located around 700 m of altitude were selected for this study (Picarrico, around 1,155 L of volume, UTM coordinates 30T 295107, 4586056 and Marlota, around 5,600 L of volume, UTM coordinates 30T 294955, 4583532). Submerged dataloggers (HOBO Pro v2 Water Temperature Logger U22-001; Onset Inc., Bourne, MA, USA) in each trough provided a continuous half-hourly measurement of water temperature from January of 2018 to January of 2019.

We visited the study area on five occasions, once every 3 months: Jan 2018, April 2018, July 2018, Oct 2018 and Jan 2019, marked individuals during the first four occasions, and collected infection samples during the last four occasions. Decontamination protocols were used between visits and nitrile gloves were used to handle individuals and were changed between sites. During each marking event we marked as many larvae as possible by injecting visible implant elastomers (VIE; North-west Marine Technology, Inc., Shaw Island, WA, USA) under the skin on the dorsal side directly in the field. A different VIE tag colour marker was used for every visit. In all cases we marked and sampled previously marked tadpoles and those unmarked individuals of a developmental stage that had not been present in the troughs in the previous visit (except for Jan 2018 for which all individuals were obviously unmarked). By doing this, our analyses were not confused by individuals that had been present in the trough at the last visit but simply missed by our sampling. That allowed us to estimate the minimum period of time that every sampled tadpole spent in water, minimizing the possible error regarding the upper period of time (except for the individuals marked during the first visit). Therefore, according to VIE marks of sampling timepoints, every sampled tadpole was classified into one category of ‘time spent in water’: 3–6 months, 6–9 months, 9–12 months, and >12 months. Tadpoles marked with two or more VIE marks were used just once in the analyses and, in all cases, just the earlier mark was considered to calculate the period of time spent in water. Additionally, during each sampling event, two or three observers independently counted every tadpole resulting in a total estimate of abundance. If one of the counts was more than twice the other, the counting was repeated, otherwise the mean count was used as the abundance. The scarcity of aquatic vegetation provides crystal-clear waters that allow precise counts.

For *Bd* diagnostic tests we gently swabbed tadpole mouthparts with a sterile cotton swab (MW113; Medical Wire & Equipment Inc., Corsham, Wiltshire, England), registered Gosner stage (by using a thread counting magnifier) and released them back into their troughs. We kept swab samples below 4 °C until processing, usually within 1–2 weeks. For the first visit in January 2018 we did not collect any pathogen diagnostic data because we had no data on any of the predictor variables for the time leading up to this first sampling occasion.

DNA extractions from the swabs were performed using PrepMan Ultra (Applied Biosystems, Foster City, CA, USA) and the amount of *Bd* DNA present in each sample was measured per duplicate through a myGo qPCR machine with a *Bd*-specific Taqman Assay (following Boyle et al., 2004). We used negative controls and synthetic oligonucleotide standards (GeneArt Strings DNA Fragment; Life Technologies Inc., Madrid, Spain) with known concentrations of *Bd* DNA copies (0.1, 1, 10, 100 and 1,000 genomic equivalents of *Bd* copies). Infection loads were assessed by the machine software according to the reference function built with the standards of known concentration of *Bd* copies. A sample was considered positive when both replicates amplified, its averaged infection load was equal to or higher than 0.1 *Bd* copies, and the amplification curve displayed a robust sigmoidal shape. If not, the sample was re-run and considered positive only with another positive result recorded.

Tadpole density was obtained by dividing the estimated abundance by the volume of the trough (that remains constant all the year around) calculated by using a rule. Following Fernández-Beaskoetxea et al. (2015), the minimum water temperatures of the 2 days previous to the sampling event were considered for further analyses, while sampling event was not because is confounded with water temperature. Pearson’ r values indicated no significant associations among tadpole density, averaged Gosner stage, and water temperatures across sampling events and cattle troughs. Water temperature and Gosner stage did not differ between both cattle troughs (*t* test, *t* ratio < 1.8, df = 6, *p* > 0.1377 in both cases), but tadpole density was higher at Picarrico trough (*t* test, *t* ratio = 3.2, df = 6, *p* = 0.0165). Therefore, tadpole density was confounded with trough and neither variables was considered together in further analyses and we could not properly test prediction P1 while accounting for the other variables.

A linear model and *post hoc* Tukey tests were used to analyse the variation in individual *Bd* loads (log transformed; *x*’ = log10[*x* + 1]) in response to our predictor variables: water temperature (P2), time spent in water (P3), and Gosner development stage (P4). Additionally, to account for the block effect of tadpoles being taken from different cattle troughs, we include the trough as a random effect too. Water temperature was included in the model as a continuous variable. Due to limitations of sample sizes, we did not include interactions between variables in our model. Homoscedasticity and normality of residuals of the final linear model was visually checked and did not show appreciable deviation from the model assumptions. Finally, although we could not include it in full modelling processes due to it being confounded with trough, tadpole density at each sampling event and cattle trough was regressed to the average *Bd* load values obtained in each case. All analyses were carried out using JMP Pro 15 (SAS Institute Inc., Cary, NC, USA).

All research was performed in accordance with relevant international guidelines and national regulations (Comité de Ética CSIC, exp. 666/2017; Comunidad de Madrid, license number for Jaime Bosch: CAP-T-0176-15). Field work was approved by the Consejería de Medio Ambiente of Castilla y León (exp. EP/CyL/97/2018).

## Results

**Figure 1:**
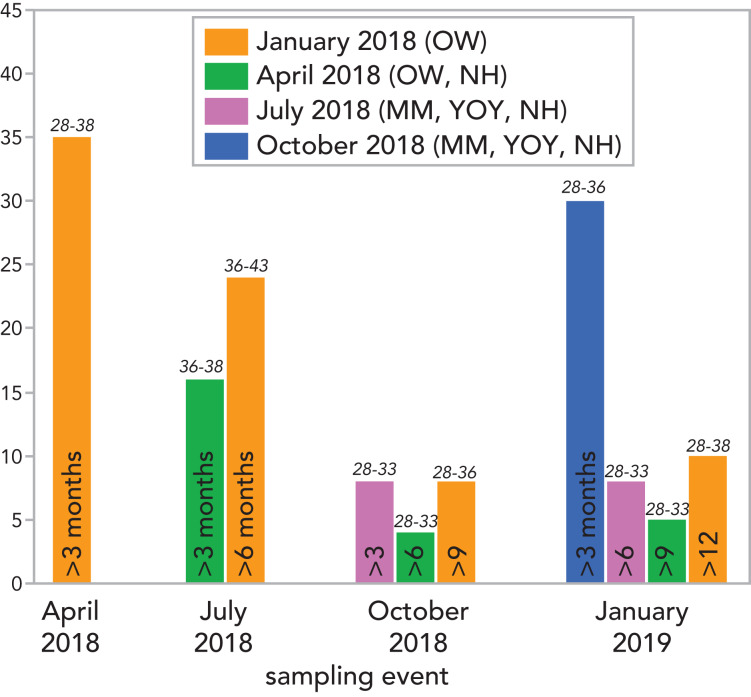
Number of *Alytes obstetricans* larvae sampled in the course of the field-study and classified into one category of ‘minimum time spent in water’ accordingly to their colour marks applied in previous marking events. The study area was visited on five occasions, once every 3 months (from January 2018 to January 2019), larvae were marked during the first four occasions, and infection samples were collected during the last four occasions. The used colour and the existing classes of larvae ages at every marking event are shown in the upper panel: OW = overwintered larvae; NH = newly hatched larvae; YOY = young of year larvae (*i.e*. no longer newly hatched, but not having overwintered), and MM = metamorphic individuals. For example, larvae marked on orange in January 2018 where classified into the category of ‘>3 months’ when found in April 2018 (*n* = 35), to ‘>6 months’ if found in July 2018 (*n* = 24), to ‘>6 months’ when found in October 2018 (*n* = 8), and to ‘>12 months’ if found in January 2019 (*n* = 10); larvae marked on green in April 2018 where classified into the category of ‘>3 months’ if found in July 2018 (*n* = 16), to ‘>6 months’ when found in October 2018 (*n* = 4), and to ‘>9 months’ if found in January 2019 (*n* = 5), *etc*. The range of Gosner development stages for each category of ‘time spent in water’ and sampling event appears in italic.

A total of 148 larvae were sampled and included into the analysis (Fig. 1). Most captured larvae had spent 3–6 months into the water, whereas just 10 spent >12 months. Prevalence of infection was almost 100% at each sampling occasion, and just one larva was not infected.

**Figure 2:**
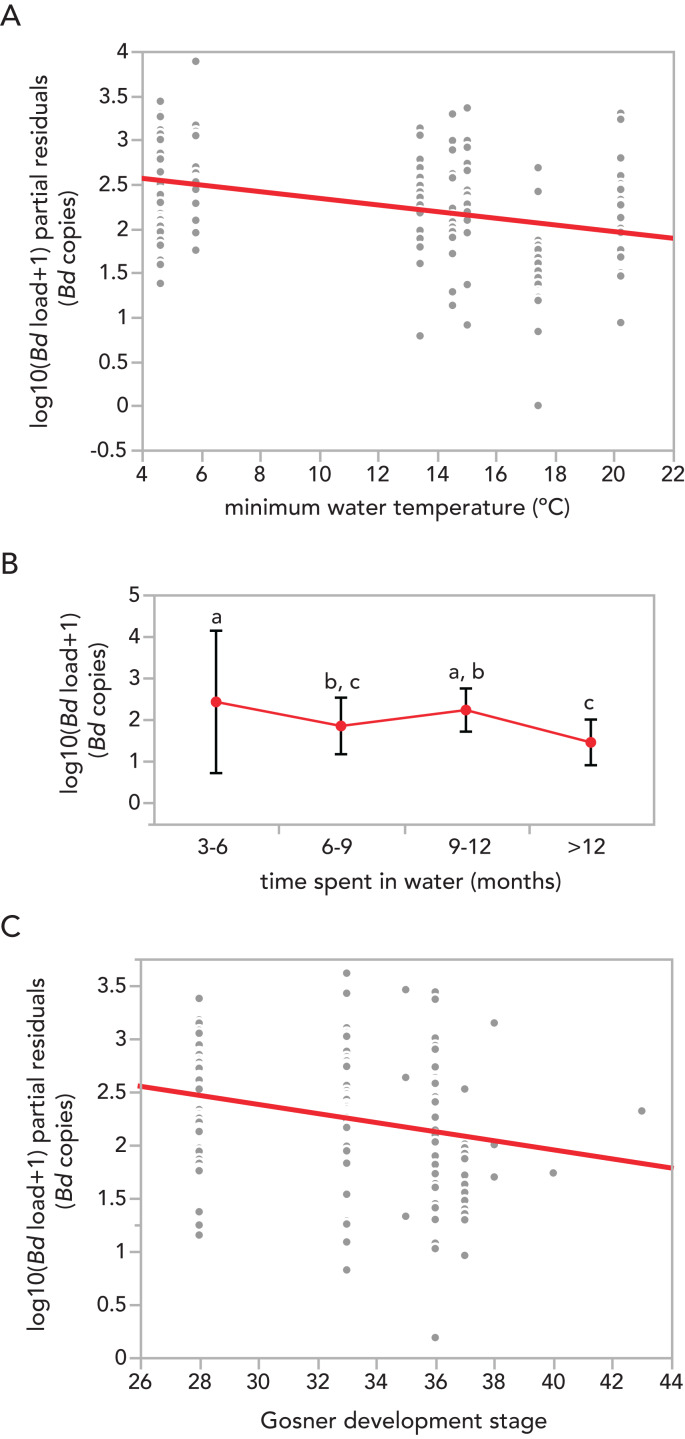
Influence of minimum water temperature 2 days before sampling, time spent in water and Gosner development stage of *Alytes obstetricans* larvae from two different sites on their *Batrachochytrium dendrobatidis* infection load (in log10(x) + 1). Residual plots (A and C) show the relationship between a given independent variable and the response, given that the other two independent variables are also in the model, thereby accounting for their effects. The lines are the partial linear regression lines. For least-squares means (B) the mean +/− std error are shown, and letters above points indicate whether levels differ according to the *post hoc* Tukey’s Honest Significant Difference test.

The model explained ~41% of the variation in infection intensity observed within the data (Table 1). F-tests suggest that the explanatory variable with the largest effect size was ‘minimum water temperature’ (F_1,142_ = 23.4, *p* < 0.001), followed by ‘time spent in water’ (F_3,140_ = 10.5, *p* < 0.001) and, with less importance ‘Gosner development stage’ (F_1,85_ = 5.4, *p* = 0.0226). As expected, water temperature was negatively correlated with *Bd* infection (Fig. 2A). According to model parameter estimates, the slope of the relationship between water temperature and infection intensity was −0.0506, which when back-transformed equates to a decrease in infection of approximately 11 zoospores per 1 °C increase in water temperature. Over the range of temperatures recorded in this study (~16 °C), this could relate to a change in infection intensity of approximately 175 *Bd* copies.

**Table 1:** Parameter estimates, standard errors, t-ratios and *p*-values for every term included into the model. Contribution of each parameter to the performed model predicting the infection load of *Alytes obstetricans* larvae. Model R^2^ = 0.412.

Term	Estimate	Std. error	*t* Ratio	*p*-value
Intercept	4.6643	0.6773	6.89	<0.0001
water temperature	−0.0506	0.0104	−4.83	<0.0001
Time spent in water:				
3–6 months	0	–	–	–
6–9 months	−0.5771	0.1391	−4.15	<0.0001
9–12 months	−0.1949	0.2093	−0.93	0.3546
>12 months	−0.9707	0.2664	−3.63	0.0004
Gosner stage	−0.0493	0.0212	−2.32	0.0226

Time spent in water and Gosner stage, unexpectedly, showed negative trends (Figs. 2B and 2C, respectively): that is, larvae spending 3–6 months in the water harboured higher infections than those that spent more than 12 months in the water (model parameter estimate of 70 *Bd* copies *vs* 29 *Bd* copies respectively, representing a decrease of 58% infection intensity). However, this relationship was non-linear, with the parameter for the 9–12 month category being higher, with a model estimate of 114 *Bd* copies per infection. Similarly, larvae at higher Gosner development stages harboured also lower infections, with an estimated decrease of less than a single *Bd* copy per Gosner stage progressed. Finally, tadpole density was not related to averaged *Bd* infection across sampling events and cattle troughs (F_1,7_ = 0.4, *p* = 0.530, *r*^2^ = 0.1).

## Discussion

Our data and analyses identify key drivers of infection intensity in individual hosts. Combined, these drivers can be used to describe a timeline by which overwintering larvae can develop and harbour high infection intensities. Larvae are most likely to adopt a life history strategy in which they overwinter in response to colder water temperatures (Morrison & Hero, 2003). In these conditions individuals may become more heavily infected as immune function may alter according to temperature (Ellison et al., 2020; Greenspan et al., 2017), while *Bd* may be locally adapted to optimise growth (Voyles et al., 2012; Stevenson et al., 2013). Density and clustering likely leads to increases in the frequency and duration of contacts with other infected individuals and also with infectious zoospores in the environment (Rachowicz & Briggs, 2007; Briggs, Knapp & Vredenburg, 2010), although our data ultimately meant we could not test this hypothesis properly. Infection intensity is ultimately limited by the finite amount of keratin in an anuran larva (*i.e*. mouthparts only). When keratin sources are depleted, more infection is impossible unless further keratin is produced, or until metamorphosis occurs and infection spreads to other keratinised parts of the body (Marantelli et al., 2004; Garner et al., 2009). Here we discuss each of these steps in turn with reference to our results.

Duration of time spent in the water body was a significant predictor of infection intensity, but in the opposite direction than expected (P3): infection intensity was highest at the shortest duration of time in the water. We expected that longer times spent in the water would result in higher levels of infection, whereas our data suggest a negative relationship between duration of time in the water and infection intensity. However, in our data larvae that had been in the water for 9–12 months had the highest predicted infection intensity, suggesting this relationship is non-linear. There are a number of possible reasons why we did observed a decline in infection intensity when and time spent in water when comparing the first and last time points. The extended period of the larval stage in species in the genus *Alytes* could lead to resource depletion for *Bd* within an individual larva. The mouthparts of infected tadpoles may become reduced in the quantity of keratin that they hold, or may degrade and entirely disappear in infected individuals (Garner et al., 2009). Either way, the net result would be a reduction in resource (keratin) available for the parasite (*Bd*) to use, and a lower infection intensity at the end time point would be the end result. If loss of keratin or entire loss of the mouthparts results in functional loss of feeding ability in larvae—those individuals may respond, as amphibian do to a host of other external stressors-with an increased rate of development (Kohli et al., 2019; Székely et al., 2017; Van Buskirk, 2016), or in death of the most compromised individuals—*i.e*. those that are most unable to feed (Garner et al., 2009; Farrer et al., 2011). In either case, the number of animals sampled at this timepoint would be lower and both the selection pressure to develop quickly and mortality rate could be higher in individuals most heavily infected with *Bd*, which would subsequently not be sampled at the last time-point. Further, the smaller number of individuals sampled at this time point relative to others (just 10 individuals out 148; Fig. 1) could result in a lower statistical power to detect variation in infection at this time point.

The Gosner stage of a larva had the same effect and directionality as that of time spent in the water; *i.e*. there was a relationship, but the directionality was not as we had expected, with higher stages (more developed) individuals having, on average, lower intensity of infection. The fact that developmental stage has a very similar relationship with infection intensity as duration in the water body makes intuitive sense—the longer an individual is in the water, the more developed it becomes. However, in some cases here the two were not confounded with each other (*i.e*. different individuals employed different life history strategies, and therefore the same Gosner stages had spent different amounts of time in the water). Despite this, the two variables exhibited a similar relationship with *Bd* copies, suggesting that both length of time and Gosner stage can both drive infection levels, likely *via* the mechanisms outlined in above. This uncoupling of Gosner stage and time spent in the waterbody highlights a novel aspect of our analyses: the presence of both in our final model suggests that it is not a case of one or the other being important, but that both play a role in explaining the patterns of infection intensity that we observed. Disentangling the relative importance of the two could provide further insights into how and why infection intensities vary within a species, with a view to understanding and managing infection levels. However, due to their close relationship altering one and not the other variable is operationally very difficult, although a more detailed, focused approach within a study system such as this one could provide future opportunities to do so.

Some of the most severe impacts of *Bd* have been recorded in montane systems where, amongst other factors, temperature tends to be lower for a given latitude. It therefore follows that the samples taken shortly after or during a period of lower water temperatures could promote higher infection intensities. Further, in this field study we used cattle watering troughs which are often fed by freshwater springs. As a result of this water source, the water temperature is often lower than might be expected at this elevational level and at the observed air temperature. As an aside, this is why measuring water temperature directly is far preferable to using air temperature-based models to predict the effects of *Bd* infection on *A. obstetricans* individuals and populations. Often water temperatures are not accurately reflected by air temperatures from meteorological stations or GIS layers because of the sources of the water-bodies in question, and the microhabitats they provide.

While water temperature, developmental stage, and duration of time spent in the water are drivers of infection, the question then becomes whether the effect sizes estimated are biologically meaningful—that is, would the size of the changes estimated make any difference to the hosts, as well as the populations and communities in which they live? Little evidence exists in most *Bd*-host systems as to how change in infection intensity relates to mortality rates or sublethal effects. Identifying hard-and-fast thresholds that are biologically meaningful is therefore difficult. What we can say is that even exposure to, rather than infection with, *Bd* can result in negative sublethal effect in species that are commonly sympatric with *A. obstetricans* (*e.g. Rana temporaria*; Bielby et al., 2015), and that the focal species of our study is important not just for itself, but for the dynamics of whole communities of amphibians (Fernández-Beaskoetxea, Bosch & Bielby, 2016; Bielby et al., 2021). Further, in other species infection status itself is not always a reliable predictor of even the most severe impact of *Bd* exposure (*i.e*. death in *Bufo bufo*; Garner et al., 2009). While this study simplified the infection dynamics considerably by only including a single species, we argue that given the complex ways in which *Bd* can affect hosts that are exposed, the patterns we have identified and the effect sizes estimated are potentially biologically informative given the role of *A. obstetricans* in the maintenance of infection, and the fact that other sympatric species may be affected even by exposure to *Bd*.

Overall, our data suggest that water temperature, developmental stage, and duration of time spent in the water body can all help to explain the patterns of infection intensity that we observe. While this adds to our knowledge of *Bd*-host dynamics, it also raises a number of knowledge gaps that may need filling if we want to better manage *Bd* as a wildlife pathogen. These gaps largely rely on us moving beyond pattern-based analyses and into the detailed mechanisms underpinning aspects of disease ecology such as the proliferation of infection within and without a host, and how these relate to transmission dynamics and ill-effects of pathogen exposure. In the absence of perfect information on these processes and mechanisms, the patterns we observe can still provide useful guidance on how, when and where mitigation strategies may be best directed.

## Supplemental Information

10.7717/peerj.12889/supp-1 Supplemental Information 1 Infection loads of *Alytes obstetricans* larvae according to their Gosner development stage, time spent in water, and density. The raw data is showing infection load, Gosner development stage, time spent in water, and density of 148 *Alytes obstetricans* larvae sampled at two sites during the four sampling events (April, July, October and January)

## Additional Information and Declarations

### Field Study Permissions

The following information was supplied relating to field study approvals (*i.e*., approving body and any reference numbers): Field work was approved by the Consejería de Medio Ambiente of Castilla y León (authorisation number: EP/CyL/97/2018).

### Data Availability

The following information was supplied regarding data availability: The raw data is available in the Supplemental File.
